# Editorial: Combating Cancer With Natural Products: What Would Non-Coding RNAs Bring?

**DOI:** 10.3389/fonc.2021.747586

**Published:** 2021-09-17

**Authors:** Yongye Huang, Yue Hou, Peng Qu, Yong Cai

**Affiliations:** ^1^College of Life and Health Sciences, Northeastern University, Shenyang, China; ^2^Cancer and Inflammation Program, National Cancer Institute (NCI), Frederick, MD, United States; ^3^School of Life Sciences, Jilin University, Changchun, China

**Keywords:** natural products, cancer, microRNA, lncRNA, circRNA

Natural products are innumerably diverse and exhibit different biological characteristics. Natural products have long been used as medicine for different applications. Chemotherapy is ranked as one of the leading cancer treatments, and a wide range of natural products are currently undergoing clinical evaluation for cancer treatment. However, there is still a large proportion of natural products that are not being used in a clinical setting. We have pointed out that managing factors in genetics, epigenetics, cancer stem cells, and the tumor microenvironment have the potential to broaden the application of natural products in cancer therapy (1, 2). Non-coding RNAs (ncRNAs) are an important component of epigenetics. Therefore, the present Research Topic aims to collect articles that evaluate the anti-tumor effect of natural products in relation to regulating ncRNAs.

The major types of ncRNAs include rRNA, tRNA, snRNA, snoRNA, siRNA, microRNA, lncRNA, and circRNA. The crosstalk among lncRNA, microRNA, and circRNA plays a key regulatory role in the progression of cancer. They can also affect cancer development and progression *via* regulating chromatin remodeling, RNA polymerase II binding to the promoter, mRNA splicing, RNA interference, protein stability and location, and more. In the Research Topic, the natural products chrysin (Chen et al.), cucurbitacin B (Tao et al.), ailanthone (Chen et al.), baicalein (Ma et al.), fucoidan (Ma et al.), casearlucin A (Li et al.), Wan-Nian-Qing prescription, bruceine D (Li et al.), and cinnamaldehyde (Chen et al.) were evaluated for their capacity to regulate ncRNAs to treat cancer (**Figure 1**). Through gathering these papers, many significant findings and implications were found.

**Figure 1:**
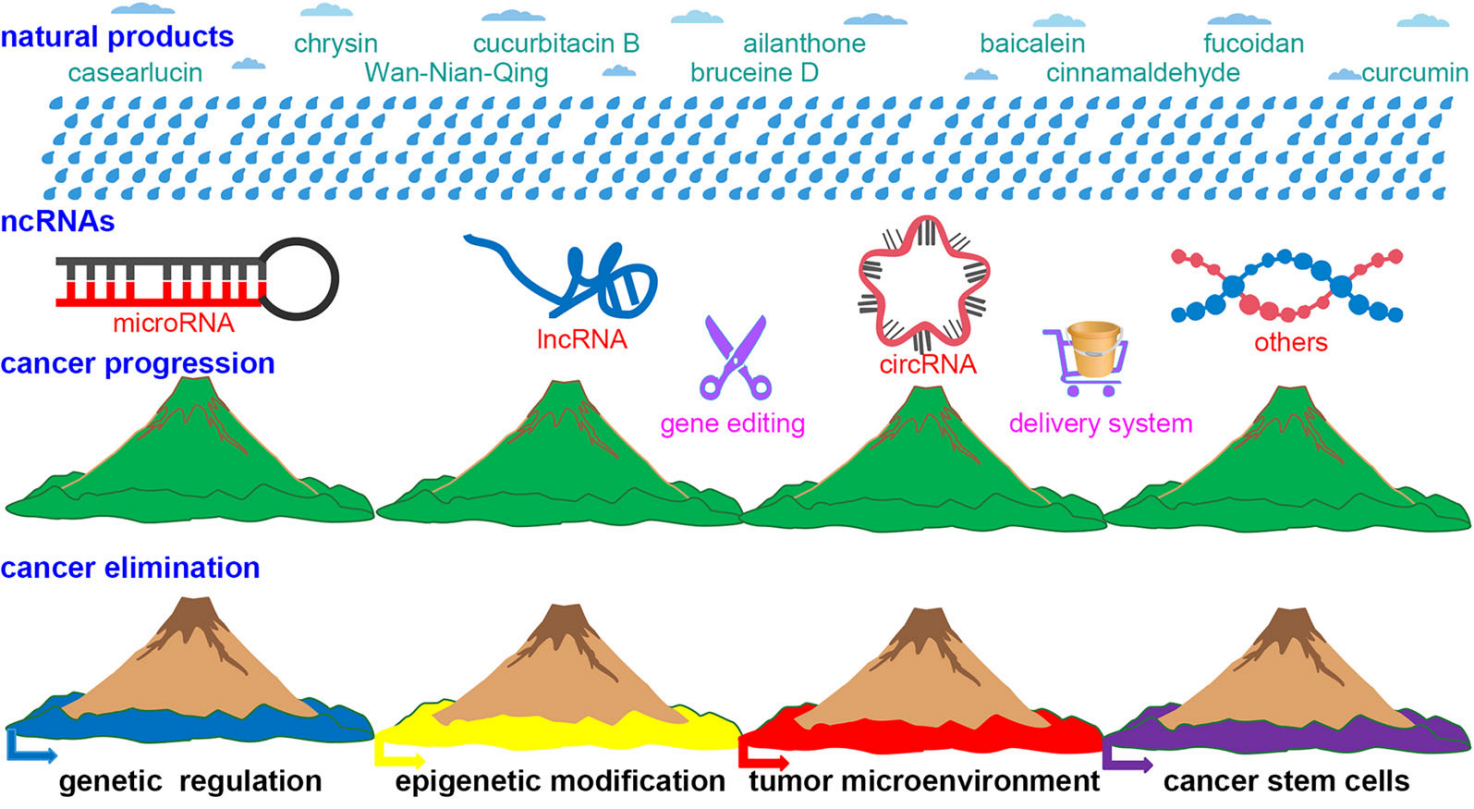
The schematic diagram of this Research Topic. Similar to the phenomenon in which rain with different chemical compositions can alter soil fertility to affect vegetation coverage, natural products with ncRNAs regulation can alter cancer cell survival rates by modulating genetics, epigenetics, the tumor microenvironment, and cancer stem cells. The gene-editing technique (i.e., Crispr/Cas9) and delivery system (i.e., exosomes) can enhance the application of natural products in cancer treatment.

## Natural Products Regulate a Certain Type of Oncogenes and Tumor Suppressor Genes

For example, p53 is shown to be upregulated by cucurbitacin B treatment to suppress cell proliferation in tongue cancer. It is well known that p53, which is of vital importance in tumor inhibition, modulates cell death and survival processes *via* regulating the expression of many anti and pro-apoptotic genes (3). Besides the p53 signaling pathway, the PI3K-Akt, JAK-STAT, Wnt/β-catenin, MAPK, and NF-κB signaling pathways can be regulated by curcumin, another natural product (Wang et al.). In addition, authors screened differentially expressed genes using an RNA-seq technique in cancer cells with chrysin (Chen et al.), ailanthone (Chen et al.), and fucoidan (Ma et al.) treatment to find many more potential target genes to expand the application of natural products.

## Epigenetic Factors are Central for Developing Anti-Tumor Applications of Natural Product

Epigenetics includes DNA methylation, chromatin remodeling, histone modification, expression of ncRNAs and RNA modification (4). As mentioned above, ncRNAs are critically important in tumorigenesis and cancer therapy. In addition, the competing endogenous RNAs (ceRNAs) network has been found to modulate gene expression, thus regulating the physical or pathological process. In this Research Topic, studies have revealed that lncRNA or circRNA functions as ceRNA with microRNAs and interacts directly with oncogenes and tumor-suppressor genes. In gastric cancer, chrysin is reported to trigger apoptosis *via* the H19/let-7a/COPB2 axis to suppress tumor growth (Chen et al.). COPB2, responsible for vesicular trafficking between the Golgi apparatus and endoplasmic reticulum, is involved in tumorigenesis in various kinds of cancer (5). In tongue cancer, cucurbitacin B inhibits cell survival *via* modulating the Xist/miR-29b/p53 axis (Tao et al.). In addition, bruceine D inhibits gastric cancer cell proliferation and chemoresistance by regulating the LINC01667/miR-138-5p/cyclin E1 axis (Li et al.). DNA methylation is another important type of epigenetic modification. The DNA methylation profile of the H19 differentially methylated region (DMR) in gastric cancer was also investigated by Chen et al. However, there are to date only a few papers addressing DNA methylation with the treatment of natural products. It would be helpful to employ precision medicine using natural products in cancer therapy by targeting such epigenetic modifications.

## Natural Products Could Be Applied to Remodel the Tumor Microenvironment

The tumor microenvironment is one of the key elements supporting cancer progression and is also a major obstacle to cancer therapy. The tumor microenvironment can modulate drug metabolism and is considered a crucial contributor to the known differential response of patients to chemotherapy (6). Natural products, such as curcumin, resveratrol, epigallocatechin gallate, phloretin, and shikonin, have shown promise in modulating tumor microenvironment (7). In addition, ncRNAs may play a critical role in this process. As revealed by Xia et al., tumor-cell and non-tumor-cell-derived exosomes carrying ncRNAs are able to modulate cancerous derivations of target cells and remodel the tumor microenvironment. As an area of concern for cancer research, the targeting of the tumor microenvironment in clinical cancer therapy should be performed by applying natural products to determine their usefulness.

## Cancer Stem Cells Could Also Be a Potential Target for Treatment With Natural Products

Cancer stem cells represent a minority subpopulation of cancer cells that possess self-renewal and differentiation abilities that drive tumorigenesis, cancer progression, and chemoresistance (8). Growing evidence has documented the close relationship between cancer stem cells and the tumor microenvironment, and many researchers have tried to determine the underlying mechanism of cancer stem cell plasticity to prevent cancer development, recurrence, and drug resistance. It is unfortunate that we did not gather papers addressing natural products targeting cancer stem cells in this Research Topic. In our previous investigation, we found that studies on this topic are not widespread (1). Some chemical modifications or techniques should be developed to improve natural products to target cancer stem cells directly and precisely in the future.

Besides obtaining theoretical advancement in this Research Topic, it also provides some useful strategies to enhance the application of natural products in cancer therapy. Exosomes are a kind of small extracellular vesicle secreted by various cell types. Exosomes are generated from late endosomes through several pathways. Most importantly, exosomes deliver various kinds of molecules, including proteins, lipids, iron, and nucleic acid. Research has indicated that exosomal ncRNAs are promising for early diagnosis and precise therapy of hepatocellular carcinoma (Xia et al.). Thus, combination with an exosomal delivery system would greatly enhance the effectiveness of natural products. Furthermore, applying gene-editing techniques in natural products-based cancer therapy could become an important strategy for anti-tumor treatment. In this Research Topic, the CRISPR/Cas9 system was used to knockout Xist in SCC9 cells to investigate the relationship between cucurbitacin B treatment and Xist expression (Tao et al.). Besides CRISPR/Cas9, A-to-I RNA editing is another advanced gene-editing technique that could be implemented in cancer treatment and investigation (Wang et al.). It would be possible to improve the anti-tumor function of natural products by applying A-to-I RNA editing to edit microRNA, lncRNA, and circRNA. Generally, the diverse research on this Research Topic provides important information for applying natural products to cancer therapy and indicates a promising direction for the future study of natural products for combating cancer in the realms of genetics, epigenetics, cancer stem cells, and the microenvironment.

## Author Contributions

All authors listed have made a substantial, direct and intellectual contribution to the work, and approved it for publication.

## Funding

YH is supported by Natural Science Foundation of Liaoning Province (2021-MS-104), and National Natural Science Foundation of China (Nos.81502582).

## Conflict of Interest

The authors declare that the research was conducted in the absence of any commercial or financial relationships that could be construed as a potential conflict of interest.

## Publisher’s Note

All claims expressed in this article are solely those of the authors and do not necessarily represent those of their affiliated organizations, or those of the publisher, the editors and the reviewers. Any product that may be evaluated in this article, or claim that may be made by its manufacturer, is not guaranteed or endorsed by the publisher.
